# Comparative Study of the Labial Gland Secretion in Termites (Isoptera)

**DOI:** 10.1371/journal.pone.0046431

**Published:** 2012-10-10

**Authors:** David Sillam-Dussès, Jana Krasulová, Vladimír Vrkoslav, Jana Pytelková, Josef Cvačka, Kateřina Kutalová, Thomas Bourguignon, Toru Miura, Jan Šobotník

**Affiliations:** 1 Institute of Organic Chemistry and Biochemistry, Academy of Science of the Czech Republic, Flemingovo náměstí 2, Prague, Czech Republic; 2 Laboratoire Écologie et Évolution, Université Pierre et Marie Curie, Paris, France; 3 Institut de Recherche pour le Développement, Unité Mixte de Recherche 211 Biogéochimie et Ecologie des Milieux Continentaux, Interactions Biologiques dans les Sols, Bondy, France; 4 Laboratoire d'Ethologie Expérimentale et Comparée, Equipe d'accueil 4443, Université Paris 13, Sorbonne Paris Cité, Villetaneuse, France; 5 Department of Analytical Chemistry, Faculty of Science, Charles University, Albertov 6, Prague, Czech Republic; 6 Department of Zoology, Faculty of Science, Charles University, Albertov 6, Prague, Czech Republic; 7 Evolutionary Biology and Ecology, Université Libre de Bruxelles, Brussels, Belgium; 8 Graduate School of Environmental Science, Hokkaido University, Sapporo, Japan; 9 Czech University of Life Sciences, Faculty of Forestry and Wood Sciences, Prague, Czech Republic; University of California Davis, United States of America

## Abstract

Labial glands are present in all castes and developmental stages of all termite species. In workers, their secretion contains a food-marking pheromone and digestive enzymes, while soldier secretion plays a defensive role. However, these functions were studied only in a limited set of species, and do not allow drawing general conclusions. Hence, we have investigated the chemical composition of the labial gland extracts from soldiers and workers in 15 termite species belonging to 6 families using an integrative approach based on proteomic and small-molecule profiling. We confirmed the presence of hydroquinone and cellulase in the labial glands of workers, and we identified new toxic compounds in soldiers and workers of several species. Our results highlight the dual role of labial gland secretion, i.e. the defensive role in soldiers and workers of several termite species, and the digestive function in workers.

## Introduction

In contrast to Hymenoptera for which about a hundred different exocrine glands are known [1], [2], only 17 exocrine glands have been described in termites [3]. Among them, labial glands (also called salivary glands) occur in all castes and developmental stages of all termite species [4], [5]. They are paired, made of numerous clumps of secretory cells (called acini) connected to the labium bases by salivary canals, to which paired reservoirs (called water sacs) are also connected.

Given the omnipresence of the labial glands in termites, there is no doubt that they play a fundamental role in the life of termite societies. On the other hand, only little is known about the chemistry of their secretion. The phagostimulating and food-marking function is filled by benzene-1,4-diol (hereafter called hydroquinone), produced by workers (hereafter used in the broad sense for working individuals in general, i.e. true workers and pseudergates) of several species [6], [7]. In lower termites and fungus-growing termites, worker labial glands are also involved in wood digestion by production of cellulolytic enzymes [8], [9], [10]. Other functions of worker labial gland secretion are only hypothetic, i.e. production of the cement used in construction and the food for the dependent castes [4], [11]. The function of the labial glands in soldiers was studied only in *Mastotermes darwiniensis* (Mastotermitidae) and several Macrotermitinae (Termitidae), in which they contribute to chemical defence by producing quinones [12], [13], [14], [15], [16], [17], mono- and sesquiterpenes, aromatic compounds or macrocyclic lactones [18], [19], [20] responsible for toxic, irritant, and congealing effects of the secretion released into the wound caused by mandibles (for review see [21]). Other partially characterized-compounds (a protein, [16]; a polysaccharide, [20]) are expected to play the role of a stiffening agent. Similar defensive purpose has also been attributed to the labial glands of workers in some soldierless termites [22]. These functional differences between castes are coupled with cell morphology [5], [23], [24], [25]; in workers, the labial gland acini are always composed of more types of secretory cells than in soldiers, in which the most abundant cell type (the only in *Macrotermes*) is structurally similar in all termite taxa [5], [24], [26], [27], [28], [29].

Soldiers and workers have distinct functions in the colony, so their glandular secretions usually dramatically differ [21]. Here, using an integrative methodological approach, i.e. a combination of Sodium Dodecyl Sulfate - Polyacrylamide Gel Electrophoresis (SDS-PAGE), Matrix-Assisted Laser Desorption/Ionisation - Time Of Flight Mass Spectrometry (MALDI-TOF), and Gas Chromatography - Mass Spectrometry (GC-MS), we compared soldier and worker labial gland secretion in 15 species, representing all major termite taxa. We also focus on the evolution of the labial gland secretion, especially the relationships between the function of the labial gland secretion and the relative development of other glands fulfilling similar roles, such as the frontal gland.

## Materials and Methods

### Insects

Table 1 lists the termite species studied and their sampling locality. After collection, termites were brought to Prague and reared in climate-controlled rooms (27±1°C, 60% RH) until dissection was made. The European population of *Reticulitermes flavipes* was formerly known as *R. santonensis*.

**Table 1:** List of studied termite species and their geographical origin.

Family	Sub-family	Species	Place of collection	n
Mastotermitidae		*Mastotermes darwiniensis*	Australia	2/2/5/5
Archotermopsidae		*Zootermopsis angusticollis*	California, USA	2/2/2/2
		*Hodotermopsis sjoestedti*	Vietnam	Not studied
Kalotermitidae		*Cryptotermes declivis*	China	7/4/5/0
		*Kalotermes flavicollis*	Italy	5/7/4/4
		*Neotermes castaneus*	Cuba	3/4/3/4
		*Neotermes cubanus*	Cuba	0/0/4/6
Hodotermitidae		*Hodotermes mossambicus*	South Africa	5/2/5/5
Rhinotermitidae	Coptotermitinae	*Coptotermes formosanus*	China	15/30/15/30
	Heterotermitinae	*Reticulitermes flavipes*	France	15/30/15/30
	Prorhinotermitinae	*Prorhinotermes simplex*	Florida, USA	15/30/15/30
Termitidae	Macrotermitinae	*Odontotermes* sp.	India	20/20/20/20
	Apicotermitinae	*Anoplotermes banksi*	French Guiana	20/-/20/-
	Termitinae	*Neocapritermes taracua*	French Guiana	15/20/5/7
		*Spinitermes* sp.	French Guiana	10/0/15/0
	Syntermitinae	*Labiotermes labralis*	French Guiana	14/9/10/10
	Nasutitermitinae	*Nasutitermes princeps*	French Guiana	20/30/20/30

n = number of workers used in extracts for SDS-PAGE/soldiers for SDS-PAGE/workers for MALDI-TOF/soldiers for MALDI-TOF.

### Dissection of the glands

Labial glands of cold anesthetized termites were dissected under stereomicroscope. Glands were transferred into distilled water for MALDI-TOF, into Tris-HCl buffer (pH 7.0) containing 0.1 M NaCl for SDS-PAGE, or into methanol for GC×GC/TOF-MS and GC-MS.

The analyses were performed on the labial gland extracts, because of the impossibility of gathering pure labial gland secretions. For each analysis, we made control samples consisting of a piece of abdomen devoid of any exocrine glands. Due to the abundant frontal gland secretion in soldiers of *Coptotermes formosanus*, which inevitably contaminates labial glands extracts, an additional control - pure frontal gland secretion - was prepared for soldiers of this species. All samples used for MALDI-TOF and GC-MS were stored in the solvent for 24 hours at 4°C, filtered and kept at −20°C until use, while samples for SDS-PAGE were homogenized with a teflon pestle homogenizer and stored at −80°C till use.

### SDS-PAGE and N-terminal protein sequencing

These methods were used to compare proteinaceous products between workers and soldiers of a given species, and among studied species. Table 1 indicates the number of workers and soldiers for each species used to prepare the extracts. These extracts were clarified by centrifugation (13,000 g, 10 min, 4°C) and the supernatants were stored at −80°C. Aliquots of the extracts were separated by Laemmli SDS-PAGE. Samples containing 10–100 µg of protein were precipitated in 80% acetone prior the analysis. The SDS-PAGE was performed on 15% gels under reducing conditions, and the gels were protein-stained with Coomassie Brilliant Blue. Proteins were electroblotted from the gel onto a PVDF membrane for N-terminal protein sequencing. N-terminal amino acid sequences were determined using a Procise 491 Protein Sequencer (Applied Biosystems) by Edman degradation. The amino acid sequences were searched in the NCBI protein database using Protein BLAST (http://blast.ncbi.nlm.nih.gov/Blast.cgi).

### MALDI-TOF

This technique was applied for comparison of the overall number of higher mass components between workers and soldiers of a given species, and among studied species. We used Reflex IV (Bruker Daltonik GmbH, Bremen, Germany) operated in linear mode with the acceleration voltage of 20 kV and 200 ns extraction pulse. Desorption and ionization was achieved using a nitrogen UV laser (337.1 nm, 4 ns pulse of 300 µJ, maximum frequency 20 Hz) with laser power adjusted to 30–35%. Matrix ions were suppressed below *m/z* 3000. Data were collected from *m/z* 3800 to 70000 and analyzed with FlexAnalysis 3.0 (Bruker Daltonik GmbH, Bremen, Germany). The mass spectra were externally calibrated using Protein Calibration Standard I (Bruker Daltonik). All spectra were averaged from 300 laser shots (10×30 shots) taken from at least 4 distinct places on a spot. For data analysis, background subtraction and smoothing were performed. Sinapinic Acid (SA) was used as a matrix. A saturated solution of SA in acetone (1 µl) was applied to the target plate and the solvent was allowed to evaporate. Sample solution (1 µl) was applied on top of the first matrix crystal layer, followed by the deposition of the second layer of the matrix from 1 µl of saturated solution of SA in ACN: 0.1% TFA in water, 1∶1. The same procedure was carried out for 2–3 samples of the same number of glands of each species and each caste (indicated in Table 1). The spectra of labial glands were compared with spectra of controls to disclose and discard the compounds coming from surrounding tissues.

### GC-MS

GC-MS analyses served for identification of the most abundant small-molecule polar compounds, and were performed on quadrupole DSQ II (Thermo Scientific) with a DB-5 column (30 m, id 0.25 mm, film thickness 0.25 µm). Temperature programme was 50°C (1 min) to 320°C (5 min) at 7°C/min. Samples in methanol were concentrated to approximately 5 µl. The number of individuals dissected for one sample differs according to species; only samples in which at least a single compound was detected are listed (for both see Table 2). When enough material was available, two dimensional gas chromatography with time-of-flight mass spectrometric detection (GC×GC/TOF-MS, Pegasus 3D, Leco) was used, as described in [30]. The temperature programme for the first column was 50°C (1 min) to 320°C (5 min) at 5°C/min and the second column was set 10°C higher. The standard of hydroquinone was purchased from Sigma-Aldrich, methanol (p.a. 99,8%) from Penta.

**Table 2:** Chemical composition of the labial gland secretion of workers and soldiers of all termite species studied by GC-MS or GC×GC/TOF-MS.

Family, sub-family	Species	Caste	Glands	Technique	Results
Mastotermitidae	*Mastotermes darwiniensis*	Workers	20	GC-MS	*p*-benzoquinone, hydroquinone, methyl glucopyranoside, *p*-arbutin
		Soldiers	20	GC-MS	*p*-benzoquinone, hydroquinone, 2-methoxyhydroquinone, methyl glucopyranoside, *p*-arbutin
Archotermopsidae	*Zootermopsis augusticollis*	Pseudergates	9	GC-MS	methyl benzoate, *p*-arbutin
		Soldiers	4	GC-MS	no compound unequivocally identified
	*Hodotermopsis sjoestedti*	Pseudergates	4	GC-MS	hydroquinone, *p*-arbutin
		Soldiers	4	GC-MS	methyl benzoate, benzoic acid, methyl 3-phenylpropanoate, 3-phenylpropanoic acid, traces of hydroquinone
Kalotermitidae	*Kalotermes flavicollis*	Pseudergates	13	GC-MS, GCxGC/TOF-MS	hydroquinone, methyl glucopyranoside, *p*-arbutin
		Soldiers	5	GC-MS, GCxGC/TOF-MS	hydroquinone, methyl glucopyranoside, *p*-arbutin
	*Neotermes castaneus*	Pseudergates	20	GC-MS, GCxGC/TOF-MS	hydroquinone, methyl glucopyranoside, *p*-arbutin
		Soldiers	6	GC-MS, GCxGC/TOF-MS	hydroquinone, methyl glucopyranoside, *p*-arbutin
	*Neotermes cubanus*	Pseudergates	23	GC-MS, GCxGC/TOF-MS	hydroquinone, methyl glucopyranoside, *p*-arbutin
		Soldiers	20	GC-MS, GCxGC/TOF-MS	hydroquinone, methyl glucopyranoside, *p*-arbutin
Rhinotermitidae	*Coptotermes formosanus*	Soldiers	100	GC-MS, GCxGC/TOF-MS	hydroquinone
Termitidae Syntermitinae	*Labiotermes labralis*	Worker	40	GC-MS	4-hydroxydihydrofuran-2(3H)-one, methyl glucopyranoside
		Soldiers	25	GC-MS	4-hydroxydihydrofuran-2(3H)-one

1–3 samples were used for each species and caste. Controls (piece of abdomen extract) were made for all samples s(Only species in which we were able to identify some compounds are listed). For *Coptotermes formosanus*, defensive secretions extract was also used as a control.

## Results

### 1. Protein profiling of labial glands

**Figure 1:**
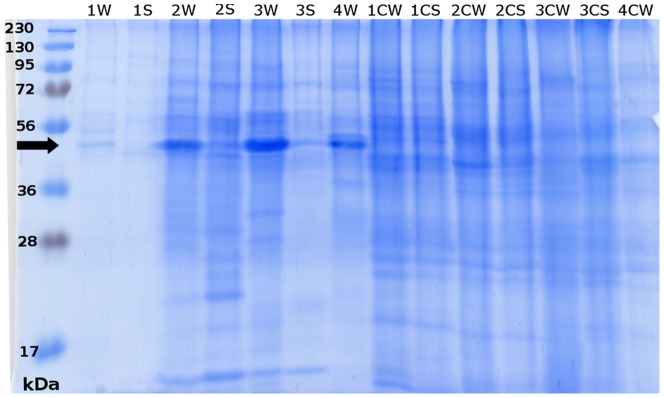
SDS-PAGE of labial gland extracts of workers (W) or soldiers (S). *Neotermes castaneus* (Nc), *Reticulitermes flavipes* (Rf), *Prorhinotermes simplex* (Ps), *Coptotermes formosanus* (Cf). Controls made of a piece of abdomen were made for each species and caste. A black arrow indicates cellulase bands.

Protein patterns of labial gland extracts were determined by SDS-PAGE. Major proteins were predominantly in the mass regions of 45, 65, and 80 kDa. Among the numerous bands visible in the labial gland extracts of *Prorhinotermes simplex*, a prominent band occurred around 45 kDa in workers (Figure 1), and was relatively less abundant in soldiers. The N-terminal sequence of the protein in workers was AYDYKKVLTNSLLFYEAQQR, which is highly homologous to N-termini of cellulases known from Mastotermitidae, Rhinotermitidae, and Nasutitermitinae (83–88% identity) (Table S1).

The band corresponding to cellulase identified in *P. simplex* was also found in workers and soldiers of *Reticulitermes flavipes*, *Coptotermes formosanus* (Figure 1), *Kalotermes flavicollis*, and *Zootermopsis angusticollis*, but not in any Termitidae sample (results not shown). Generally, this protein was more abundant in workers compared to soldiers. A weak cellulase band was also present in workers of *Neotermes castaneus* but is probably absent in soldiers (Figure 1).

For workers of *Neocapritermes taracua*, the major band was around 80 kDa (Figure S1) and its N-terminal sequence was identified as KPLPISRLQDDLKEFMELVPTDKILEIT. This band was not present in the protein pattern of soldiers. Based on the sequence homology (53–70% identity), this protein is related to class 1 allergens from cockroaches such as Bla g 1 from *Blatella germanica*, which were found in various insects (Table S2). The same compound could also be present in workers and soldiers of *Labiotermes labralis* (Figure S1).

The N-terminal sequences AMPRIISTSYGGA and XPLPIAKFTQELLN were identified from a band of 20 kDa in soldiers of *R. flavipes* and from a band of 70 kDa in workers of *Nasutitermes princeps*, respectively, but no homology could be found to sequences available in the sequence databases.

### 2. MALDI-TOF

Specific and reproducible peaks observed in workers and soldiers are listed in Table 3. Among all the peaks visible in extract of *P. simplex* workers, the only reproducible and specific peak was of m/z 47088, roughly corresponding to the mass of cellulase, identified in SDS-PAGE. A peak of m/z close to 47088 was also observed in workers of *R. flavipes*, *C. formosanus*, *N. castaneus*, and in workers and soldiers of *K. flavicollis*. These peaks likely correspond to cellulase. The various species of termites synthesized different molecules of cellulase with slightly different m/z. Cellulase identified by SDS-PAGE in many species was not found by MALDI-TOF in all these species, especially in soldiers (Table 3), supposedly because of the suppression of ionization by other compounds in the solutions. Compounds of identical m/z were found in *K. flavicollis*, *N. castaneus*, and *C. formosanus* (Table 3). Both workers and soldiers of *Mastotermes darwiniensis* showed many compounds in the m/z range 24189–32227. Only compounds of low m/z ratio were determined in *N. princeps* workers and soldiers. Other compounds were present in many species while no specific peak was observed in *Neocapritermes taracua* and *L. labralis*. An identical situation occurred in soldiers of *C. formosanus*, *R. flavipes* and in workers of *Odontotermes* sp. (Table 3).

**Table 3:** Reproducible peaks (X) obtained by MALDI-TOF in labial glands extracts in termite workers (w) and soldiers (s). Missing species and castes represent samples devoid of reproducible peaks.

Species	Caste																		
		6067	6866	8369	9969	10680	11321	13433	17216	17940	18385	19412	23164	23485	23507	23543	23594	23632	23779
*Mastotermes darwiniensis*	w												X						
	s												X						
*Zootermopsis angusticollis*	w																		X
	s																		
*Kalotermes flavicollis*	w															X			
	s									X									
*Neotermes castaneus*	w						X									X			
	s				X			X											
*Neotermes cubanus*	w				X									X					
	s																X		
*Cryptotermes declivis*	w																		
*Hodotermes mossambicus*	ww																		
	bw																		
	s																	X	
*Coptotermes formosanus*	w															X			
*Reticulitermes flavipes*	w														X				
*Prorhinotermes simplex*	w																		
*Odontotermes* sp.	s						X												
*Nasutitermes princeps*	w			X		X			X		X	X							
	s	X	X																

																
24189	24347	24532	24717	24916	32227	46966	47000	47028	47088	47105	47140	47191	47270	47472	47531	64384
X	X	X	X		X									X		X
X	X	X	X	X												
															X	
														X		
									X							
								X								
										X						
																
						X										
																
						X										
											X					
													X			
												X				
										X						
							X									
									X							

A distinction has been made in *Hodotermes mossambicus* between white workers (ww) and black workers (bw).

### 3. GC-MS, GC×GC/TOF-MS

**Figure 2:**
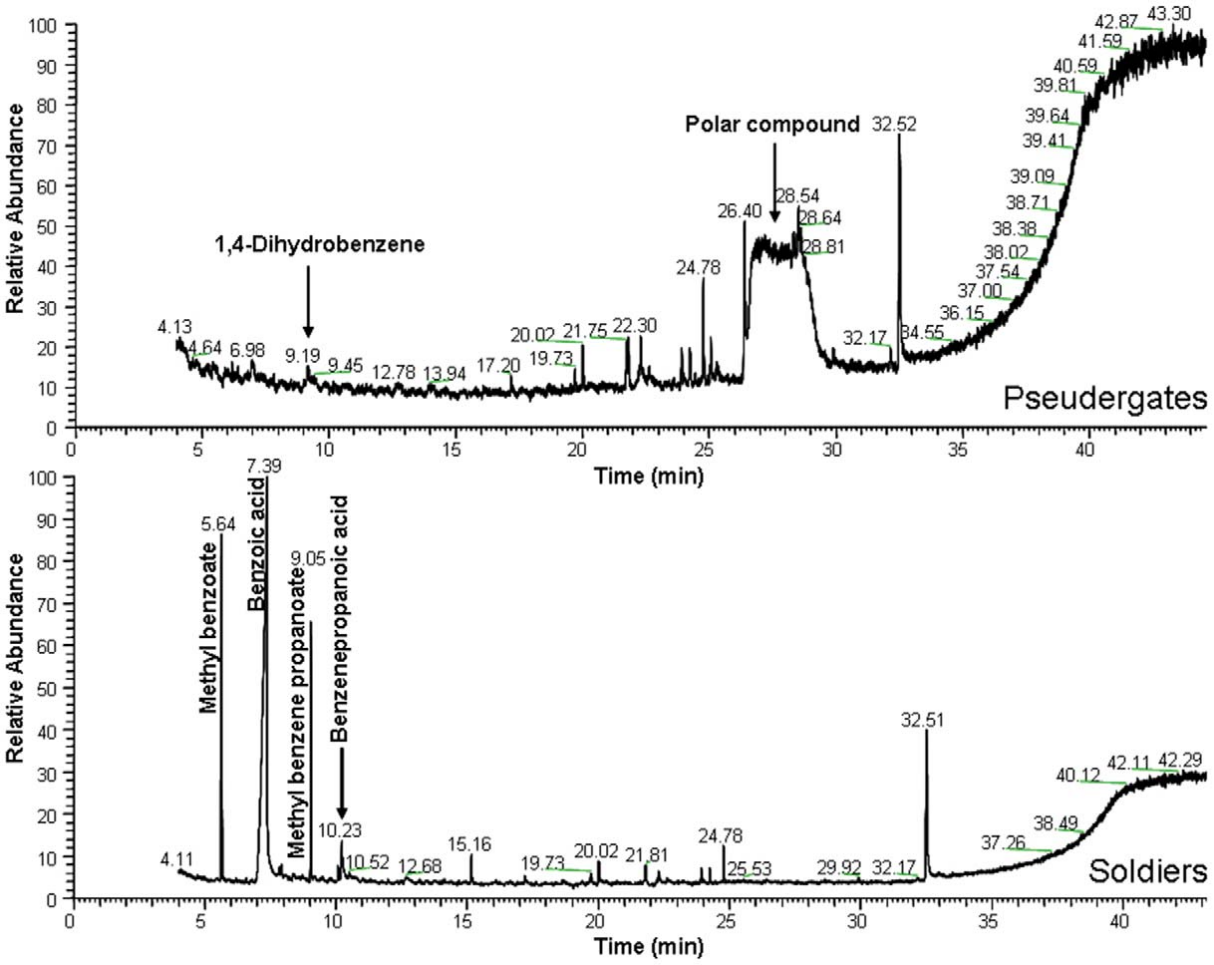
Comparison of GC-MS profile of labial gland extracts between pseudergates and soldiers in *Hodotermopsis sjoestedti*.

Table 2 summarizes the compounds identified by these techniques. We confirmed the presence of hydroquinone in the labial glands of many species. The other compounds presented in the majority of species were *p*-arbutin and methyl glucopyranoside. Furthermore *p*-benzoquinone and 2-methoxyhydroquinone were found in workers and/or soldiers in *M. darwiniensis*. Interestingly, the labial glands of soldiers in *Hodotermopsis sjoestedti* contained benzoic acid, 3-phenylpropanoic acid and their corresponding methyl esters in addition to hydroquinone (Figure 2). The other compound (4-hydroxydihydrofuran-2(3H)-one) was identified in both castes of *L. labralis* (Table 2).

## Discussion

This study focuses on the chemical composition of the labial gland secretions in workers and soldiers of 15 termite species belonging to different families and sub-families (comprising all termite families except for Stolotermitidae and Serritermitidae). The use of several techniques allowed the identification of compounds of different properties (polarity, m/z ratio, molecular weight), as well as a comparison of the dis/similarities between castes.

For long it was believed that cellulose digestion in termites is ensured by symbiotic gut microorganisms, until Watanabe and co-workers [31] unequivocally demonstrated that termites can produce their own cellulases and do not necessarily rely upon their symbionts to process cellulose. Cellulases are produced in the labial glands and/or midgut of workers and probably soldiers [9], [10], [32], [33]. By protein microsequencing, we identified cellulase in the labial glands of the workers of *P. simplex*, a species not studied yet for this aspect. Moreover, strong evidence of cellulase production was found by both SDS-PAGE and MALDI-TOF in labial glands of *P. simplex* soldiers, *N. castaneus* workers, and both castes of *R. flavipes*, *C. formosanus*, *K. flavicollis*, and *Z. angusticollis*. Ultrastructural observation of labial glands in workers and soldiers of *P. simplex* revealed the presence of the same type of protein-producing cells (called type IIa) [5], which are probably responsible for cellulase production. Our results also confirm the previous studies [9], [10] showing that in the so-called “lower” termites, cellulases are produced by the labial glands and are more abundant in workers than in soldiers. Similarly to these results, we confirmed the absence of cellulases in labial glands of Termitidae, in which its production is restricted to the midgut [9], [10].

The labial glands are connected to the mouth by two canals though which the secretions flow is the mouth and can then be swallowed (in the case of digestive enzyme produced by workers), applied on food source (as a food-marking pheromone in workers), mixed with other materials used in building activities performed by workers, provided to other colony members when workers feed the dependent castes, or spit on opponents during combat. Such diverse functions of the labial gland secretion in workers would probably not be performed by the same compounds, so mechanisms of regulation of the composition of the labial gland secretion are expected. Such regulation seems possible, according to observations of axons located below the basement membrane of acini (see e.g. [5], [26], [27]) and supposedly innervating particular secretory cells.

Hydroquinone has been identified in the secretions of the labial glands of workers in several termite species [7]. Here, we confirmed its presence in some of them (*M. darwiniensis*, *K. flavicollis*, *C. formosanus*) and also found it in labial glands of workers of a few other species (*H. sjoestedti*, *N. castaneus*, *N. cubanus*), as well as in soldiers of all the above-mentioned species. Apart from the role of hydroquinone in food marking [7], it is known as allomone from defensive secretions of many insects (for review see [34]) and millipedes [35]. It is not surprising as its toxicity is well-documented (for details see e.g. [36]). In the majority of species, we detected *p*-arbutin (glycosylated hydroquinone) which could be hydrolyzed by *β*-glucosidase to hydroquinone [37], [38]. Moreover, it appears possible that hydroquinone is a precursor of *p*-benzoquinone as it can be oxidized under mild conditions to produce *p*-benzoquinone [39]. *p*-Benzoquinone has been identified in the soldiers' labial gland secretion of *Mastotermes* [12] (see also Table 2), *Macrotermes* [13], *Hypotermes* [14], and *Odontotermes* [16]. It is known to be an irritating compound and is frequently found in defensive secretions of insects and other Arthropods (for review see [34], [40]). In addition to hydroquinone and *p*-benzoquinone, the labial gland secretion of *M. darwiniensis* soldiers contains 2-methoxyhydroquinone which was found in defensive secretion of tropical millipedes [41]. In *H. sjoestedti*, several interesting compounds have also been identified in addition to hydroquinone: benzoic acid and 3-phenylpropanoic acid, both known as defensive compounds from pygidial glands of Dytiscidae beetles [42], methyl benzoate found as the major poison gland product of ant *Messor barbarus* [43] and methyl 3-phenylpropanoate which was found in honeybee propolis [44]. Methyl benzoate has also been found in *Z. angusticollis* workers. Moreover, the other compound has been identified in the labial glands of both castes in *L. labralis*, 4-hydroxydihydrofuran-2(3H)-one, previously detected in extracts of the fungus *Trametes* (Basidiomycota: Polyporaceae) with antifungal activities [45].

A food-marking role of this gland that stimulates gnawing and feeding has already been described in workers of several species [6], [25], [46], [47], [48], [49]. It appears possible that the small molecules from the labial gland secretion may, at least in some species, have a dual role in workers, food-marking and defence. The defensive role of these glands in workers remains to be demonstrated with bioassays, but several arguments such as the toxic compounds and the stiffening secretion produced in several species support this idea [21], [22]. Even though the soldier caste is the caste dedicated to the defence by its morphological and behavioural adaptations, workers play an active role in defence in many species [50], [51] and may even possess specialized defensive organs, such as the dehiscence glands in *Ruptitermes* or *Neocapritermes taracua* [52], [53]. The labial glands could reinforce the action of the workers for the protection of their colony and some observations tend to indicate that such situation indeed occurs in soldierless termites [22]. At last, the dual role of hydroquinone has been demonstrated for food [7] and for repellent action in *C. formosanus* [54] depending on the concentration used.

Besides being toxic and serving a defensive role, another important action of the secretion is its stiffening after exposure to the air. It is reported by many authors [4], [12], [22], but more details are provided only about a partially characterized protein in *Odontotermes* [16] and a polysaccharide in *Pseudacanthotermes* [20], which are supposedly responsible for the stickiness of the total secretion.

It is clear that the secretion of labial glands in termites is not limited to a few compounds. Only the dominant compounds have been identified by means of gas chromatography and the detection capacity of the technique does not allow the identification of minor compounds supposedly present in the secretion. Valuable information about the chemical composition of labial glands can be obtained using different techniques. We identified a few more compounds but MALDI-TOF technique showed clearly that the secretion is made of a blend of many compounds, with minor compounds supposedly reinforcing the action of the major ones.

We are also facing important difficulties due to methodological constraints. The secretion of labial glands is always water-carried, thus particular compounds are in general polar and many of them are probably not vaporizable at GC-MS conditions. Another factor that should be taken into account is that the soldiers' defensive compounds are either produced by the frontal and/or by the labial glands, and one of the glands is always dominant. It seems clear that soldiers of basal families (Mastotermitidae, Hodotermitidae, Archotermopsidae, Stolotermitidae, Kalotermitidae *sensu* [55], lacking the frontal gland, rely only upon labial glands, while in more advanced families (Rhinotermitidae, Serritermitidae, Termitidae), having both glands, it is usually the frontal gland, which is the dominant defensive organ. The only exception occurs in soldiers of Macrotermitinae (Termitidae), in which enlarged labial glands [23] play a prime role [13], [14], [16], [17], [20] while the frontal gland produces small amounts of secretion with anti-healing properties [56]. In our study, the only identified defensive compound produced by the labial glands in soldiers having the frontal gland as a dominant defensive organ, is hydroquinone found in *Coptotermes formosanus*. Several hundred pairs of labial glands were necessary to confirm the presence of hydroquinone in *C. formosanus*, an unmanageably high number for other species. Moreover, the inevitable contamination of samples by the frontal gland products makes the work more difficult.

## Supporting Information

Figure S1 SDS-PAGE of labial gland extracts of workers (W) or soldiers (S). *Coptotermes formosanus* (Cf), *Neocapritermes taracua* (Nt), *Labiotermes labralis* (Ll). Controls made of a piece of abdomen were made for each species and caste. A black arrow indicates cellulase bands. A white arrow indicates class 1 allergen bands.
(TIF)

Table S1 Comparison of the N-terminal amino acid sequence of the cellulase determined for *Prorhinotermes simplex* workers with homologous sequences from other termite species (GenBank accession numbers are indicated).
(DOC)

Table S2 Comparison of the N-terminal amino acid sequence of a class 1 allergen-like protein determined for *Neocapritermes taracua* workers with homologous sequences from other insect species (GenBank/NCBI accession numbers are indicated).
(DOC)
