# Correction: Comparative Study of the Labial Gland Secretion in Termites (Isoptera)

**DOI:** 10.1371/annotation/c56eb874-fb41-4d90-9735-b84d593f32cd

**Published:** 2012-11-14

**Authors:** David Sillam-Dussès, Jana Krasulová, Vladimír Vrkoslav, Jana Pytelková, Josef Cvačka, Kateřina Kutalová, Thomas Bourguignon, Toru Miura, Jan Šobotník

There was an error in Figure 2. The correct Figure 2 can be viewed here: 

**Figure:**
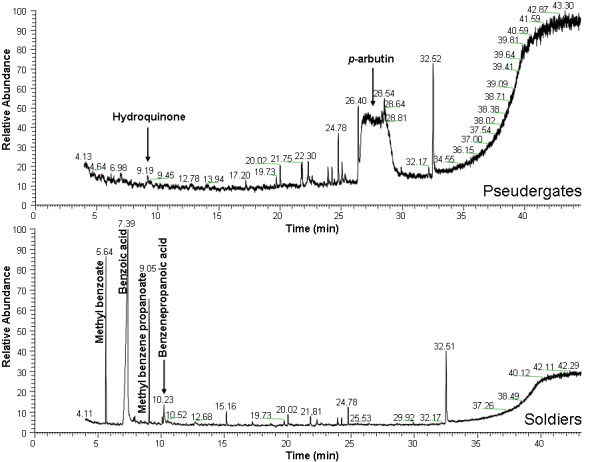


.

