# Supplementary figures and images for: Comparative Study of the Labial Gland Secretion in Termites (Isoptera)
Source: PLoS One. 2012 Oct 10;7(10):e46431. doi: 10.1371/journal.pone.0046431 (PMC3468581; doi:10.1371/journal.pone.0046431)

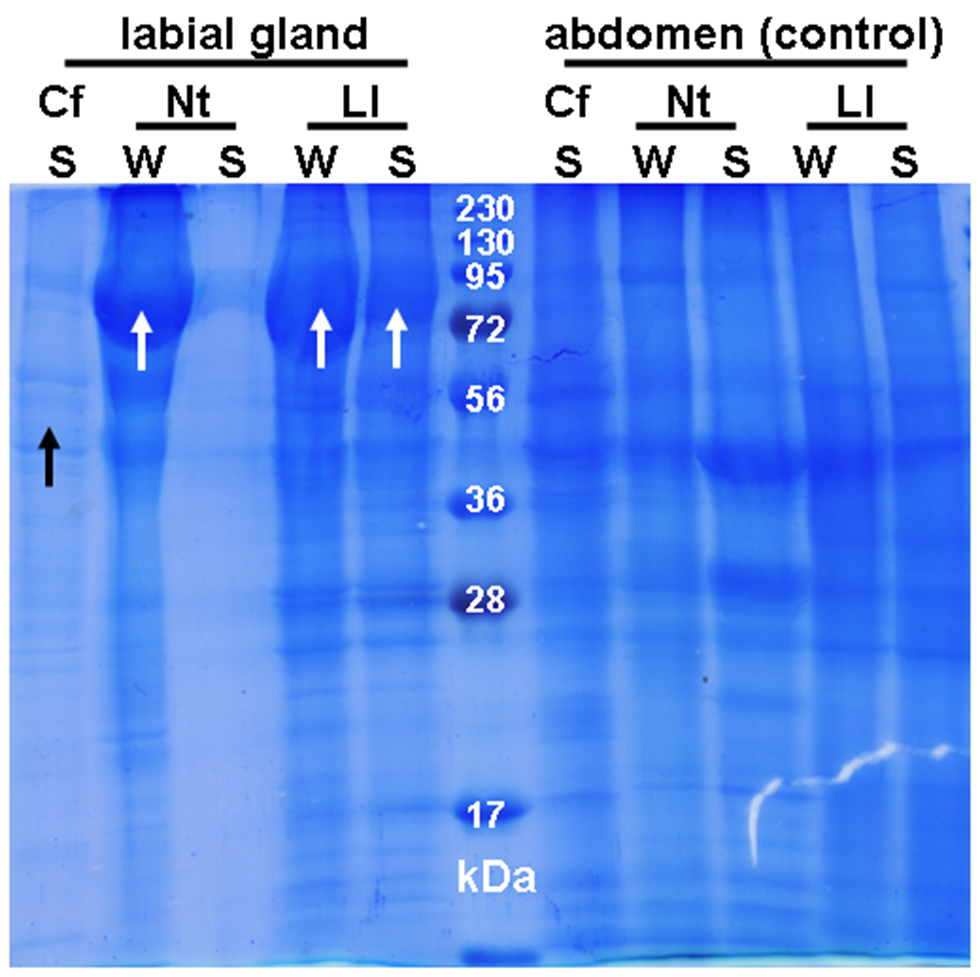

Supplement: Figure S1 — SDS-PAGE of labial gland extracts of workers (W) or soldiers (S). Coptotermes formosanus (Cf), Neocapritermes taracua (Nt), Labiotermes labralis (Ll). Controls made of a piece of abdomen were made for each species and caste. A black arrow indicates cellulase bands. A white arrow indicates class 1 allergen bands. (TIF) [file pone.0046431.s001.tif]
