# Supplementary material for: Comparative Study of the Labial Gland Secretion in Termites (Isoptera)
Source: PLoS One. 2012 Oct 10;7(10):e46431. doi: 10.1371/journal.pone.0046431 (PMC3468581; doi:10.1371/journal.pone.0046431)
Supplement: Table S1 — Comparison of the N-terminal amino acid sequence of the cellulase determined for Prorhinotermes simplex workers with homologous sequences from other termite species (GenBank accession numbers are indicated). (DOC) [file pone.0046431.s002.doc]

Table S1. Comparison of the N-terminal amino acid sequence of the cellulase determined for *Prorhinotermes simplex* workers with homologous sequences from other termite species (GenBank accession numbers are indicated).

| **Species** | **Description** | **Sequence** | | | | | | | | | | | | | | | | | | | | **Accession number** |
| --- | --- | --- | --- | --- | --- | --- | --- | --- | --- | --- | --- | --- | --- | --- | --- | --- | --- | --- | --- | --- | --- | --- |
| *Prorhinotermes simplex* |  | A | Y | D | Y | K | K | V | L | T | N | S | L | L | F | Y | E | A | Q | Q | R |  |
| *Reticulitermes flavipes* | Endogenous cellulase | A | Y | D | Y | K | T | V | L | S | N | S | L | L | F | Y | E | A | Q | R | S | AAU20853 |
| *Reticulitermes speratus* | Endoglucanase 2 | A | Y | D | Y | K | T | V | L | S | N | S | L | L | F | Y | E | A | Q | R | S | BAA34050 |
| *Reticulitermes speratus* | Salivary cellulase | A | Y | D | Y | K | T | V | L | S | N | S | L | L | F | Y | E | A | Q | R | S | BAA28815 |
| *Mastotermes darwiniensis* | β-1,4-endoglucanase | A | Y | D | Y | K | D | V | L | T | K | S | L | L | F | Y | E | A | Q | R | S | CAD54729 |
| *Coptotermes formosanus* | Endo-β-1,4-glucanase | A | Y | D | Y | K | T | V | L | K | N | S | L | L | F | Y | E | A | Q | R | S | BAB40697 |
| *Nasutitermes walkeri* | Endo-β-1,4-glucanase | A | Y | D | Y | K | Q | V | L | R | D | S | L | L | F | Y | E | A | Q | R | S | BAA33709 |
| *Nasutitermes takasagoensis* | Endo-β-1,4-glucanase | A | Y | D | Y | K | Q | V | L | R | D | S | L | L | F | Y | E | A | Q | R | S | BAA33708 |

Residues identical with those of *P. simplex* are shaded in grey.
