# Supplementary material for: Comparative Study of the Labial Gland Secretion in Termites (Isoptera)
Source: PLoS One. 2012 Oct 10;7(10):e46431. doi: 10.1371/journal.pone.0046431 (PMC3468581; doi:10.1371/journal.pone.0046431)
Supplement: Table S2 — Comparison of the N-terminal amino acid sequence of a class 1 allergen-like protein determined for Neocapritermes taracua workers with homologous sequences from other insect species (GenBank/NCBI accession numbers are indicated). (DOC) [file pone.0046431.s003.doc]

Table S2. Comparison of the N-terminal amino acid sequence of a class 1 allergen-like protein determined for *Neocapritermes taracua* workers with homologous sequences from other insect species (GenBank/NCBI accession numbers are indicated).

| **Species** | **Description** | **Sequence** | | | | | | | | | | | | | | | | | | | | | | | | | | | | | **Accession number** |
| --- | --- | --- | --- | --- | --- | --- | --- | --- | --- | --- | --- | --- | --- | --- | --- | --- | --- | --- | --- | --- | --- | --- | --- | --- | --- | --- | --- | --- | --- | --- | --- |
| *Neocapritermes taracua* |  | K | P | L | P | I | S | R | - | L | Q | D | D | L | K | E | F | M | E | L | V | P | T | D | K | I | L | E | I | T |  |
| *Culex quinquefasciatus* | G12 | S | P | A | A | G | R | A | - | L | Q | D | D | L | N | E | F | V | E | L | L | P | V | D | K | I | I | D | I | V | XP_001847069 |
| *Periplaneta americana* | Allergen | G | L | T | R | A | A | R | N | L | Q | D | D | L | N | D | F | L | A | L | I | P | T | D | Q | I | L | A | I | A | AAD13533 |
| *Periplaneta americana* | Allergen | T | L | S | S | G | Q | R | N | L | Q | D | D | L | N | D | F | L | A | L | I | P | T | D | Q | I | L | A | I | A | AAC34736 |
| *Periplaneta americana* | Allergen | K | S | L | P | - | N | R | N | L | Q | D | D | L | N | D | F | L | A | L | L | P | V | D | E | I | T | A | I | V | AAB82404 |
| *Periplaneta americana* | Allergen | L | T | R | E | A | - | R | N | L | Q | D | D | L | N | D | F | L | A | L | I | P | T | D | Q | I | L | A | I | A | AAC34737 |
| *Periplaneta americana* | Allergen | G | L | T | R | A | A | R | N | L | Q | D | D | L | N | D | F | L | A | L | I | P | T | D | Q | I | L | A | I | A | AAC34312 |
| *Lutzomyia longipalpis* | Microvillar-like protein | S | T | G | V | V | Q | R | G | L | Q | D | D | L | K | E | F | V | D | L | I | P | M | N | K | I | M | S | V | A | ABV60297 |
| *Blattella germanica* | Allergen | - | - | - | - | - | S | R | N | L | Q | D | D | L | Q | D | F | L | A | L | I | P | V | D | Q | I | I | A | I | A | AAD13532 |
| *Blattella germanica* | Allergen | L | N | A | K | A | S | R | N | L | Q | D | D | L | Q | D | F | L | A | L | I | P | V | D | Q | I | I | A | I | A | AAD13530 |

Residues identical with those of *N. taracua* are shaded grey.
